# Improving Death Literacy Through Interdisciplinary Research-Practice Partnerships and Experiential Learning

**DOI:** 10.1093/geroni/igaf122.2096

**Published:** 2025-12-31

**Authors:** Kelly Melekis, Kelly Melekis

## Abstract

Death literacy is considered both an outcome of one’s experiences of and learning about death and dying, and a resource for strengthening individual and community capacity for end-of-life care. In this symposium, a group of interdisciplinary faculty will share ways they have utilized research-practice partnerships and experiential learning to improve death literacy. The symposium will begin with an overview of death literacy and the development of a multi-site collaboration to enhance death literacy through 1) research practice partnerships between community-run social model hospice homes and thanatology researchers, and 2) a Community Action, Research, and Education (CARE) experiential learning program whereby participants care for hospice patients in their last months of life. The second presentation will highlight opportunities available to students at a rural liberal arts college, discussing the potential for building death literacy and social capital for enhancing community capacity for end-of-life care. In the third presentation, a prior CARE fellow will share her experience of developing death literacy through a community-based participatory research (CBPR) project and present the results of a mixed methods study of hospice patients’ experiences with hyperactive delirium in the last week of life. A fourth presentation will describe CBPR and the use of an expansive qualitative caregiver narrative database to develop stage plays aimed at improving death literacy. An excerpt will be presented along with strategies for dissemination and community engagement. The symposium will conclude with a discussion of ways to improve death literacy through interdisciplinary research-practice partnerships and experiential learning.

